# Dental Physiology

**Published:** 1863-09

**Authors:** W. H. Atkinson


### DENTAL PHYSIOLOGY,

BY W. H. ATKINSON.

This subject can have no complete announcement without at the same time giving expression to the principles of the basis, and purpose of all organic function.

As the highest life actions are foreshadowed in the simplest bodies, and the lowest functions repeated in the highest and most complicated corelation of organs; the teeth may be said to embody the simplest and subserve the highest functions of human physiology, the first in the mechanical comminution of food, and the second in the divine expression of thought resultant upon a well fed system by *speech*. A correct report of Dental Physiology should set forth a synopsis of every possible function performed by teeth.

It is manifest that this herculean labor is far in the advance, not only of any living man's single powers, but also of the aggregate details of all observers which have been noted and placed upon record.

A lucid synopsis of what is known, and acknowledged to be the proper function of teeth, will go very far to prove our lack of knowledge in that direction and may serve to stimulate the spirit of research which is some day to be blessed with clear apprehensions of the significance of types of teeth, as indications of legitimate uses to which they may be put by their possessors. The means of keeping the teeth of human beings in a physiological state are few and very easy of textual or aphoristic statement, however difficult it may prove to induce the degenerate offspring of a luxurious race, to yield implicit obedience to the laws governing their being.

Habits are denominated good or bad as they tend to the full development, for vigorous use of every organ and every faculty, or to their deterioration in size, power and efficiency.

In view of this statement, it will not be difficult to designate the *character* of the *habits* of the American people as a whole, which have so interferred with the development of the teeth, that we have scarcely an individual in the community, who could be safely cited as an example of a purely *physiological* dental function.

How may we best secure a physiological state to our teeth?

*Ans.*—The methods of procedure promptly and completely to effect this desirable end, would also be the methods of securing uninterrupted health to the whole organism. But a few rules well observed, will be a good beginning in the work of establishing a true dental physiology.

I. Never expectorate, but swallow all the secretions of the mouth.

II. Eat regularly at stated periods, and take no intermediate lunches.

III. Never eat when very weary until some drink has been swallowed—pure water is best.

IV. Never eat to depletion nor exercise violently immediately after.

V. Keep your teeth and the surface of your body scrupulously clean.

VI. Avoid taking any substance into the system which is not properly a food.

VII. Exercise freely but regularly every day, and take all the sleep you can in one uninterrupted effort in the night, (darkness).

He who has never suffered a twinge of pain knows nothing of the value of immunity therefrom, and can have no real sympathy with those who are called to suffer for the infraction of law in themselves, progenitors, or confreres; and hence when pain does at last overtake him he is unmanned at once, and is in a pitiable state of excitement and apprehension consequent upon his ignorance of suffering and its consequences. All his boasted stamina and capability for endurance vanishes at the first demand for the proof of their possession, and he is proved to be much the inferior of the feeble sufferer, who has been crucified again and again in the furnaces of pathological experiences, and can now calmly and intelligently administer to his relief by exercising the knowledge thus attained.

So we may say he who has never known other than purely physiological states of mind and body, is a novice, and really more intensely demands our condolence and sympathy than those who have passed the baptism of frequent crucifixions of the affections and tortures of physical suffering.

The great use of the study of physiology, being to teach us how to avoid pathological conditions, it is next thing to impossible to confine ourselves to an undisturbed functional activity, or purely antegrade physiological role of organic movement; seeing that the dark back ground of departure therefrom so environs us on all sides that the antithesis is ever with us in either latent or active state.

Then if we can not in the nature of things *now*, have other than theoretical knowledge of a pure dental physiology, let us endeavor to accept and thoroughly investigate the highest approximation thereto that is within present reach, not resting satisfied to be as well posted as our progenitors and elder brethren only, but push our observations and studies deeper and deeper into the unexplored recesses of life actions, in small or great fields of her occult and open labors, as we may find opportunity to gratify our thirst, to become familiar with and able to interpret her every act.

Clearly to apprehend the proper physiology of the component parts of teeth, it becomes necessary for us to reach back into the histogeny thereof, for without perfect germs completely nourished, we can have no pure physiology in the bodies produced.

If we attempt to deduce a doctrine of perfection of organic correlation of elements, and parts from the writings extant upon this subject, we shall soon become convinced, that confusion and vague attempts at explanation, hold the dominion in this border land, connecting the seen and unseen bodies, whose workings together constitute what is nominated function; the incomplete balance of which excludes the bodies from the normal pale of physiological activity.

In the main they all tacitly or expressly adopt the atomic theory of origin to elemental bodies; but in attempts at explanation and close definition, the terms, molecule, granule and cell are so confounded as to preclude us from deciding which is the primal body.

The term *cell* is used in a multiplicity of senses covering not only the idea of simple, but compound elementary bodies, and even tissues if not organs. Let me state, then, that a simple, proper, primal cell is a *seen demonstrable* body, with a wall and contents, while a molecule, granule or atom is always an *unseen*, and therefore, *not* demonstrable body, by any other than purely theoretic processes.

The great mistake has been the failure to see that all bodies in their reality and specificity of character exist in the unseen before they can put on the symbolic, seen or the or the so called "real" material or "actual" presence; and any imperfection of the elements ef "type," necessarily lay the foundation of that state of function called pathological, as certainly and effectually, as does any disturbance of the law of balance of equivelancy, in the blastema or plasm, out of which bodies are evolved under the control of that type.

If the type then be in only a pathological condition itself, would it be reasonable to hope for a body physiologically endowed, to be produced under its incomplete dominion.

Having premised then, that all our physiology on this planet, is but a higher or less divergent pathology, the light begins to break in upon us, teaching us that all our definitions are but approximate not absolute.

However teeth being the organs which connect the rest ot the system with that part of the kingdom of nature deemed inorganic, we may fairly expect to meet in them with nearer approximation to the purely physiological state; as the lower we descend in the scale of life endowment, the less complicated will we find the elements of bodies, and consequently fewer deflections from the normal standard in number and degree will present themselves.

The old definitions of the three kingdoms of nature so called, viz: "Stones, (minerals), *grow*, vegetable *live* and *grow*, animals *live*, *grow* and *feel*,"—will answer for very general and vague investigators only, as growth is as legitimately an act of affinity or life in its degree, as are either other of the modifications of the same presence denominated living and feeling.

There must needs be affinity of some sort and some degree to permit even the relation of contact in bodies simple or compound, primal or ultimate. So we argue that the intimacy of contact,—not to say interblending (as the microscope reveals), of enamel and dentine, of this and cement on the one hand, or dentine and pulp on the other, without inciting incongruity of action in either, from the lowest to the highest, or the highest to the lowest in life endowment, we must come to the conclusion, that it is at least, a similar if not identical presence, which holds the dominion of each and renders each subservient to the other, and the whole subservient to the entire system to which it belongs.

Fact and experience prove what theory here suggests, viz: that enamel is the most perfect of all the dental tissues, and more nearly approaches uniformity of type, wherever this tissue is distinctly pronounced. Just as we rise from the simple primatism of enamel rods, do we meet with increased departure from uniformity of structure, which renders it doubtful under what category of tissue to set it down, until we have conclusively proven the mode of nutrition and comparative age of the tissue.

Vicarious performance of function is no less displayed in teeth than in the neural and vascular or glandular systems. Where there are but two tissues composing the tooth, viz: pulp and dentine, (or its cartilagenous representative), we discover a superior degree of calcification, hardening the outer portions of teeth liable to exposure of contacts with hard substances, to a degree but little inferior to enamel, whose function it performs remarkably well.

Upon this principle of vicarious hardening, was based the practice of the older dentists, of filing out the decay and polishing the exposed dentine of human teeth, which was such a blessing where calcific action was induced, and such a burning approbrium where it failed to be set up to arrest the progress of the disintegration of the now exposed dentine, which refused to do vicarious duty from inability to deposit calcareous salts in the peripheral ends of the tubules, and thus simulate the structure for which it was forced to stand.

Another vicarious action in both enamel and dentine has attracted some attention under the heads of "tooth edge," "tenderness," &c., &c., in which these dense structures have performed the function of nerve; (the most remarkable anomaly in the whole economy, if indeed, the mineral enamel is inorganic).

The query whether this is a physiological or pathological act, will be answered by reference to what was said on a foregoing page of absolute and approximate definitions.

Where contacts are so violent as to produce pain, the majority would be inclined to the term pathologic, while by those on whom it was only able to announce its presence, by their simply feeling it, the physiological character would be claimed.

My own conviction is that all well organized human dentine *is sensitive* to the cuts of an excavator, when in a state of our best examples of health until after calcific obliteration of the tubules, when it can be cut with impunity equal to sound enamel; and hence I can easily conceive the action to be both vicarious and truly physiological, as it comes within the range of instantaneous extinction by spontaneous systematic, or premeditated chemical, or electrical change of the neural current.

Just how far vicarious function can modify the physical character and office of teeth, and other bodies within the limit of a strict physiology has not been demonstrated. But that modifications to very considerable degree do take place, the varieties of color, shape and term of life of organs and persons, branching from an identical stock or parentage abundantly testify; for if type alone held the dominion, every tooth and every body would be like the parent type, in exact accordance with the degree of development, age, sex and other typal conditions.

But as type and use or function seem to divide the perfectibility of teeth and other bodies, it ought to appear to the philosophical mind, that where enamel rod continued to perform the function of nerve for a series of generations, it must lose more and more of its hardness, and adapt itself little by little to the predominent conditions necessary to more perfect performance of its adopted functions.

As to the query of whether function originates modifications of the type of the various forms of classes and individual teeth? or the type predetermines form, size, complications of structure, other character and functions of teeth? it is of the utmost importance for us to know the correct solution, before we can assuredly determine and differentiate the normal from the vicarious and the physiological, from the pathological functions of these intensely interesting and instructive, so deemed simple bodies!

Doubtless use modifies development within the limits of type, while type classifies and distributes, to their proper location the various forms of teeth, wherever she finds means to fulfill her work; so that there is a mutual dependence of the one upon the other for the complete expression of either concurrent, democratic, molecular and autocratic, systemic or typal, accord alone being able to hold the dominion in the republic of molecules whose community constitutes bodies of complete development.

Any thing short of perfect symbolization of type being but a side choice of nutriment and growth, by which pathological conditions insinuate themselves, and thereby produce adventitious varieties of sickly bodies, which mar the type by unduly nourishing one set of tissues or organs, and repressing by atrophy others as necessary to the system, thus distorting and debilitating the whole!

Having grouped together all the acts of creation, evolution and use pertaining to the teeth, we may easily apprehend the liability there is to imperfectly effect the work in the varous stages and degrees to perfection, amid the multiplicity of forces, each of which clamors to subdue to his exclusive control all the others! This anarchy among molecules runs riot in the vast majority of dental systems in human mouths in America! And I am sorry to be forced to acknowledge, that something very much like a similar anarchy holds the rein in our dental physiology.

But that a better day may speedily dawn upon us, all that is necessary is to have each one set about the work of redemption, from ignorance and the sins of an imperfect practice consequent thereupon, with all his might, determined not to rest contented, short of complete satisfaction to his own perception of every query that spontaneously, or suggestively presents itself for solution.

How may we then best avoid continuance or repetition of this anarchy, or pathological status of our doctrinal and professional physiology?

*Ans.* By each acting fully up to his individual capacity, with direct reference to communicating the work of isolation, that it may shed its light upon the aggregate professional body.

To do this faithfully and fully is the only way by which the whole can be harmoniously correlated in demonstrations clear and certain. And this can not be effected until a more earnest, self-sacrificing spirit shall become prevalent among us.

However well a few may fulfill these requirements, our progress in true knowledge will be but a lame or pathological expression, until the individual members of our body, like the molecules of living physical bodies, shall *all* fulfill their specific work of detail, in the complete harmony of consentaneous action of support to the whole, or be, like effete molecules and cells purged from the commuity of the system.

